# Randomized, double‐blind, 6‐week non‐inferiority study of lurasidone and risperidone for the treatment of schizophrenia

**DOI:** 10.1111/pcn.12965

**Published:** 2020-01-08

**Authors:** Yuan Feng, Jianguo Shi, Lili Wang, Xia Zhang, Yunlong Tan, Jingyuan Zhao, Yuping Ning, Shiping Xie, Xuejun Liu, Qi Liu, Keqing Li, Xiaoliang Wang, Lehua Li, Xiufeng Xu, Wei Deng, Xiaoyan Luo, Gang Wang

**Affiliations:** ^1^ Department of Psychiatry, Beijing Anding Hospital, National Clinical Research Center for Mental Disorders Capital Medical University Beijing China; ^2^ Department of Psychiatry Xi'an Mental Health Center Xi'an China; ^3^ Department of Psychiatry Tianjin Anding Hospital Tianjin China; ^4^ Department of Psychiatry Wuxi Mental Health Center Wuxi China; ^5^ Department of Psychiatry Beijing Huilongguan Hospital Beijing China; ^6^ Department of Psychiatry Henan Provincial Mental Hospital Xinxiang China; ^7^ Department of Psychiatry Guangzhou Brain Hospital Guangzhou China; ^8^ Department of Psychiatry Nanjing Brain Hospital Nanjing China; ^9^ Department of Psychiatry Brain Hospital of Hunan Province Changsha China; ^10^ Department of Psychiatry Sixth Hospital of Peking University Beijing China; ^11^ Department of Psychiatry Hebei Mental Health Center Baoding China; ^12^ Mental Health Institute, Shanghai Mental Health Center Shanghai Jiao Tong University School of Medicine Shanghai China; ^13^ Department of Psychiatry Second Xiangya Hospital of Central South University Changsha China; ^14^ Department of Psychiatry First Affiliated Hospital of Kunming Medical University Kunming China; ^15^ Department of Psychiatry West China Hospital of Sichuan University Chengdu China; ^16^ Medical Division Sumitomo Pharma(Suzhou)Co., Ltd Beijing China

**Keywords:** atypical antipsychotics, lurasidone, non‐inferiority trial, risperidone, schizophrenia

## Abstract

**Aim:**

The aim of the present study was to evaluate the efficacy and safety of lurasidone for the treatment of Chinese schizophrenic patients.

**Methods:**

Hospitalized schizophrenia patients aged 18–65 were randomized to 6 weeks of double‐blind, double‐dummy, flexible‐dose treatment with lurasidone (40 or 80 mg/day) or risperidone (2, 4 or 6 mg/day). Efficacy was evaluated using a non‐inferiority comparison of lurasidone relative to risperidone based on week 6 change in the Positive and Negative Syndrome Scale (PANSS) total score. Safety assessments included adverse events, clinical laboratory measures, and electrocardiograms.

**Results:**

Four hundred and forty‐four patients were screened to obtain an intent‐to‐treat sample of 384 patients, of whom 54 patients discontinued treatment prior to 6 weeks. Lurasidone met the criteria for non‐inferiority *versus* risperidone on the PANSS total score. Adjusted mean (SE) change at week 6 on the PANSS total score was −31.2 (1.0) and −34.9 (1.0) in the lurasidone and risperidone group, respectively. The mean difference score was 3.7, and the upper boundary of the 95%‐confidence interval (1.0–6.3) was less than the prespecified margin of 7.0. No clinically meaningful between‐treatment group differences were evident on secondary efficacy measures, including PANSS positive, PANSS negative, Clinical Global Impression scale – Severity, and Calgary Depression Scale for Schizophrenia scales. The incidence of adverse events was lower for lurasidone vs risperidone for extrapyramidal symptoms (17.0% vs 38.2%), akathisia (7.2% vs 13.6%), prolactin increase (3.1% vs 14.1%), and weight increase (0.5% vs 5.2%).

**Conclusion:**

Lurasidone was found to be non‐inferior to risperidone on the primary endpoint with minimal effects on weight, metabolic parameters, or prolactin levels.

Schizophrenia is a disabling psychiatric disorder characterized by hallucinations, delusions, disorganized thinking, negative symptoms, disorganized or abnormal behavior, and/or impaired executive functioning. The severity and chronicity of the disorder is associated with substantial impairment in functioning, increased healthcare utilization, and increased morbidity and mortality, including risk for suicide.^1^, ^2^, ^3^, ^4^, ^5^, ^6^, ^7^, ^8^

Lurasidone is an atypical antipsychotic approved in 2010 for the treatment of schizophrenia in the United States, with subsequent approvals in the European Union and other countries. The mechanism of action of lurasidone appears related to its potent antagonist affinity for dopamine D_2_ and serotonin 5‐HT_2A_ and 5‐HT_7_ receptors.^9^ Lurasidone also exhibits moderate affinity as an antagonist at α_2A_ and α_2C_ adrenergic receptors, and partial agonist affinity at 5‐HT_1a_ receptors.^9^ The pharmacokinetics of lurasidone are dose‐proportional within a range of 20–160 mg/day.^10^ With daily dosing, steady‐state concentrations are reached within 7 days.

Short‐term efficacy and safety of lurasidone in the treatment of schizophrenia has been demonstrated in five placebo‐controlled 6‐week trials.^11^, ^12^, ^13^, ^14^, ^15^ Evidence for longer‐term efficacy has been demonstrated based on a 12‐month non‐inferiority study comparing lurasidone to quetiapine XR^16^ and a 28‐week placebo‐controlled withdrawal study.^17^ A 12‐month, randomized, double‐blind safety study found minimal impact on body weight and prolactin for lurasidone compared to risperidone.^18^ Network meta‐analyses showed that although there might be not significant differences in the efficacy outcomes between lurasidone and risperidone, lurasidone seems to be safer than risperidone with respect to several adverse events such as cardiometabolic risk and increased prolactin.^19^, ^20^, ^21^

The objective of the current study was to evaluate the short‐term efficacy and safety of lurasidone for the treatment of schizophrenia among patients in China. Risperidone was chosen as the atypical antipsychotic comparator in the non‐inferiority study reported here because its efficacy in the treatment of schizophrenia is well‐established, and because it is among the most frequently prescribed atypical antipsychotics, both worldwide, and in China.^22^, ^23^, ^24^ There is a twofold rationale for studying the efficacy and safety of lurasidone in the Chinese population. First, there appears to be a significant cross‐cultural difference in the prevalence and patterns of diagnosis of schizophrenia in China compared with the United States and Europe, suggesting that the schizophrenia phenotype may differ sufficiently to warrant a confirmatory clinical trial.^25^ Second, there is evidence suggesting differences in CYP450 metabolic enzymes in Chinese populations that might have an impact on the tolerability and/or efficacy of antipsychotic medication.^26^, ^27^, ^28^

## Methods

### Study design

This was a multicenter, randomized, flexible‐dose, double‐blind, double‐dummy, 6‐week non‐inferiority study comparing the efficacy and safety of lurasidone to risperidone in the treatment of schizophrenia. The study was conducted at 16 investigational sites in China, with data collection occurring from January 2014 to April 2015. Following a screening visit, hospitalized patients who continued to meet study entry criteria after completion of a 7‐day washout period were randomized, in a 1:1 ratio, to 6 weeks of treatment with lurasidone (40 or 80 mg/day) or risperidone (2, 4, or 6 mg/day). Patients could remain in the hospital setting for the duration of the double‐blind treatment period, but patients considered by the investigator to be clinically stable could be discharged after 3 weeks.

Study medication was provided as identically matched (in color, shape, size, and packaging) lurasidone or risperidone tablets. An interactive web response system used a computer‐generated list of random numbers to allocate study treatments. None of the investigators, study staff, or patients had access to the randomization codes or list during the study. Because of the difference in titration schedule for lurasidone and risperidone, a double‐dummy design was utilized to ensure that the double‐blind was maintained: patients randomized to lurasidone also received a risperidone‐matched placebo, and patients randomized to risperidone received a lurasidone‐matched placebo.

Doses for both lurasidone and risperidone were administered orally once daily after dinner. The starting dose of lurasidone was 40 mg/day. After 6 days, the dose could be increased to 80 mg/day based on the patient's symptoms and tolerability. Risperidone was initiated at 2 mg/day. After 3 days, the dose was increased to 4 mg/day for 3 days. On day 7, the dose was increased to 6 mg/day or maintained at 4 mg/day, based on the patient's symptoms and tolerability. For both lurasidone and risperidone, the dose had to be increased to the higher dose at weeks 2, 3, or 4 if change from baseline on the PANSS was less than nine points (unless significant adverse events were present). The dose could be reduced at any time if intolerable adverse events occurred.

The study was approved by an Ethics Committee at each investigational site, and written informed consent was obtained from all patients prior to any study procedures. For patients younger than age 20, written informed consent was also obtained from the patient's legal guardian. The study was conducted in accordance with the International Conference on Harmonization Guideline for Good Clinical Practice and the Declaration of Helsinki.

### Patients

Eligible for the study were patients 18–65 years of age meeting the Diagnostic and Statistical Manual of Mental Disorders, Fourth Edition, Text Revision edition (DSM‐IV‐TR) criteria for a primary diagnosis of schizophrenia. In addition, patients needed to have at both screening and baseline a score ≥ 4 on the Clinical Global Impression‐Severity scale^29^ and a Positive and Negative Syndrome Scale (PANSS)^30^ total score of ≥70 and ≤120, with a score of 4 (moderate) or higher on 2 or more items of the following PANSS items: delusions, conceptual disorganization, hallucinations, unusual thought content, and suspiciousness. Key exclusion criteria included any current clinically significant neurological, metabolic (including type 1 diabetes), hepatic, renal, hematological, pulmonary, cardiovascular, gastrointestinal, or urological disorders; HIV; alcohol abusers or alcohol dependency; long QT syndrome or required a drug that treats arrhythmia; prior history of poor clinical response and/or lack of tolerability to risperidone.

### Efficacy assessments

The primary efficacy variable was the total score on the 30‐item clinician‐rated PANSS. Secondary efficacy variables included PANSS subscales (positive, negative), the Clinical Global Impression‐Severity of Illness (CGI‐S) and Clinical Global Impression‐Improvement scale (CGI‐I),^29^ and the Calgary Depression Scale for Schizophrenia (CDSS).^31^ Responder criteria consisted of ≥20% endpoint improvement in the PANSS total score. Efficacy measures were administered at screening, baseline, and at post‐baseline weeks 1, 2, 3, 4, and 6 (or study termination), with the exception of the CGI‐I, which was also administered at post‐baseline visits. In the present study, inefficacy was defined as worsening psychosis.

### Safety and tolerability assessments

Safety assessments included incidence of adverse events (AEs), serious adverse events (SAEs), vital signs, electrocardiograms (ECGs), body weight, laboratory tests (including HbA1C, lipids, prolactin, hematological indices), and urinalysis. AEs, body weight, and vital signs were assessed at every study visit (baseline, weeks 1, 2, 3, 4, and 6). ECGs and laboratory tests were performed at baseline, week 3, and week 6 (or last visit, if the patient discontinued prematurely).

The clinician‐rated Barnes Akathisia Scale,^32^ Abnormal Involuntary Movement Scale (AIMS),^30^ and Simpson Angus Scale (SAS) (Simpson and Angus, 1970)^33^ scales were used for evaluating akathisia, abnormal movement, and Parkinsonism, respectively. These scales were administered at baseline and weeks 1, 2, 3, 4, and 6.

### Concomitant medication

As‐needed treatment was permitted for movement disorders, preferably, benzhexol (up to 15 mg/day); alternatively, biperiden (up to 16 mg/day) or diphenhydramine (up to 100 mg/day), could be used. Treatment with propranolol (up to 120 mg/day) or amantadine (up to 300 mg/day) was permitted as needed for akathisia. As‐needed use of benzodiazepines (lorazepam, zolpidem, and zopiclone) was permitted for severe anxiety, agitation, or insomnia. Anxiolytics, sedatives, or hypnotics were not to be administered within 12 h prior to an assessment visit.

### Statistical analysis

The intent‐to‐treat (ITT) efficacy analysis population was defined as all randomized patients who received at least one dose of study drug/medication and had at least one post‐baseline PANSS efficacy evaluation. A per‐protocol analysis population was defined as the subset of the ITT population that had been treated for 14 days or longer, had received 75% to 125% of study treatment, and had no major protocol violations. The safety population consisted of all randomized patients who received at least one dose of study medication.

The primary efficacy endpoint was change from baseline to week 6 on the PANSS total score for the ITT sample. Non‐inferiority of lurasidone relative to risperidone was evaluated by comparing the upper boundary of the 95% confidence interval (CI) to a pre‐specified non‐inferiority margin of a difference of 7.0 PANSS total score points. A mixed model repeated measures (mmrm) method, including all weekly visits, was used to estimate the change to week 6. The mmrm included terms for treatment group, visit, center, baseline PANSS total score, and the treatment by visit interaction. An unstructured covariance matrix was specified for the within‐patient correlation, and the Kenward‐Rogers approximation was used for determining the denominator degrees of freedom. Because of potential bias when using an ITT sample to examine non‐inferiority, it has been recommended to examine non‐inferiority using a per‐protocol sample.^34^ Accordingly, a supportive mmrm analysis was also conducted on the per‐protocol sample for testing non‐inferiority. In addition to the mmrm, a supportive analysis for the primary efficacy variable was conducted within the ITT sample using an analysis of covariance (ancova) model, with the last observation carried forward (LOCF) method applied for patients with no measurement at week 6. In the ancova model, change from baseline to endpoint was set as the response variable and treatment, center, and baseline PANSS total scores were set as independent variables. Effect sizes were calculated using the Cohen's d statistic.

Secondary efficacy variables were also analyzed within the ITT sample using an mmrm approach similar to that conducted on the primary endpoint, except that standard superiority testing was conducted rather than inferiority testing. For the CGI‐I, no baseline score was included in the model. Confidence intervals (CI) around the estimated difference between the lurasidone and risperidone groups were calculated to evaluate whether the CI did not include zero (indicating superiority). Supportive ancova models (LOCF) were also performed on the secondary variables. Responder status was analyzed using logistic regression with treatment group as a categorical factor and baseline PANSS total score as a covariate. No adjustments were made for multiple comparisons.

ancova models (LOCF) with treatment, center, and relevant baseline scores were used to analyze the change from baseline to endpoint on the following safety variables: BARNES total score, AIMS total score, and SAS total score. Non‐parametric rank anova (LOCF) was used to analyze selected laboratory analytes, body mass index (BMI), and body weight. The analysis of all other safety variables was descriptive.

The target sample size was based on statistical power analysis for the non‐inferiority comparison of lurasidone and risperidone on the primary endpoint and incorporated the pre‐specified margin and estimates of the common standard deviation (SD) and attrition. The non‐inferiority margin of 7 points on the PANSS total score was based on the International Conference of Harmonization E9 guideline (International Conference on Harmonization E9 Expert Working Group)^34^ and was similar to that used in previous trials with risperidone in which the margin has ranged from 6 to 7.3.^35^, ^36^, ^37^ A common SD of 20 on change on the PANSS total score was derived from a previous lurasidone clinical trial. Using these values together with an estimated discontinuation rate of 20%, the total sample size was calculated to be 380 patients (190 per group) to have 85% power at the 2.5% significance level.

For safety analysis, the occurrence rates of adverse events were compared using the χ^2^ test or Fisher's Exact test as appropriate. Number needed to harm (NNH) values were calculated as the reciprocal of the difference in the prevalence of each adverse event.

## Results

### Patient characteristics and disposition

**Figure 1:**
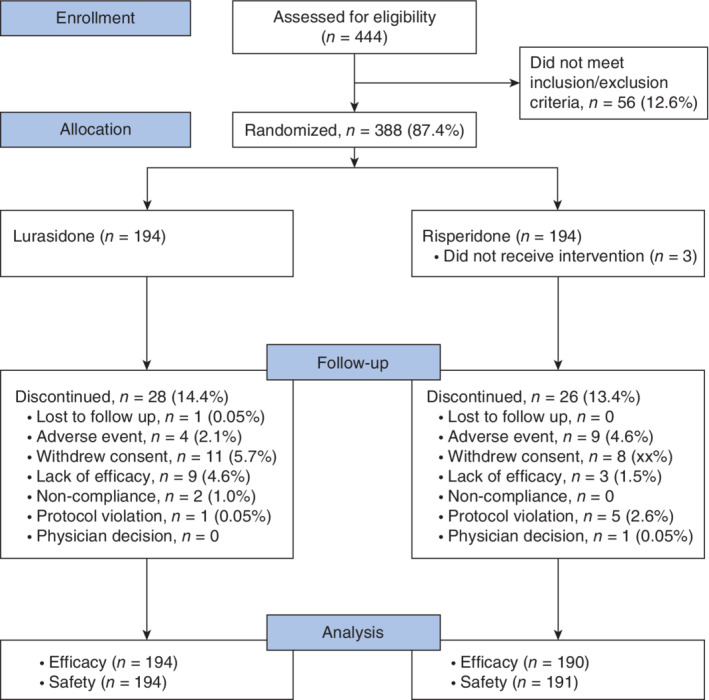
Patient disposition.

There were 444 patients screened for the study; of these, 388 (87.4%) were randomized (194 in each treatment group; Fig. 1). In the risperidone group, three patients did not receive the study drug and were excluded from the safety analysis population, and four patients did not have a post‐baseline assessment and were excluded from the intent‐to‐treat (ITT) population. A total of 54 patients (13.9%) discontinued treatment prior to 6 weeks (28 (14.4%) in the lurasidone group; 26 (13.4%) in the risperidone group). The median days of exposure to study drug for the safety analysis population was 42 days for each treatment group, with approximately 90% of patients receiving ≥29 days of study treatment (89.7% for lurasidone; 90.1% for risperidone).

The ITT population was approximately 50% male, with a mean age of 35 years (Table 1). The majority of patients were diagnosed with paranoid‐type schizophrenia (58.9%), followed by undifferentiated type (40.9%). No notable differences were observed between the treatment groups in baseline characteristics.

**Table 1:** Patient demographic and clinical characteristics (ITT population)

	Lurasidone (*n* = 194)	Risperidone (*n* = 190)
Age, mean (SD), years	34.6 (11.1)	34.8 (10.7)
Male, *n* (%)	98 (50.5)	101 (53.2)
BMI (kg/m^2^), mean (SD)	23.3 (4.4)	23.0 (4.1)
Subtype of schizophrenia, *n* (%)		
Paranoid	119 (61.3)	107 (56.3)
Residual	1 (0.5)	0
Undifferentiated	74 (38.1)	83 (43.7)
Prior antipsychotic medication, *n* (%)	150 (77.3)	158 (83.2)
Years since onset of first episode, mean (SD)	8.12 (7.95)	8.45 (8.82)
Years since onset of current episode, mean (SD)	1.61 (3.85)	1.54 (3.94)
PANSS total score at baseline, mean (SD)	90.7 (9.7)	91.5 (9.5)
CGI‐S score at baseline, mean (SD)	5.3 (0.7)	5.4 (0.6)
CDSS total score at baseline, mean (SD)	1.1 (2.3)	0.9 (1.9)

BMI, body mass index; CDSS, Calgary Depression Scale for Schizophrenia; CGI‐S, Clinical Global Impression scale – Severity; PANSS, Positive and Negative Syndrome scale.

### Efficacy

Results of the primary (mmrm) efficacy analysis on the ITT population found that lurasidone met *a priori* criteria for non‐inferiority to risperidone (Table 2). The mean between‐treatment group difference in endpoint PANSS change scores was 3.7 (95%‐CI, 1.0–6.3). Criteria for non‐inferiority were met because the upper boundary of the 95%‐confidence interval (6.3) was less than the pre‐specified margin of 7.0. Criteria for non‐inferiority were also met based on an analysis of the per‐protocol population, with a mean between‐treatment group difference of 3.7 points (95% CI: 1.1, 6.4).

**Table 2:** Change in efficacy measures from baseline to week 6 (ITT population)

Measure	Lurasidone (*n* = 194)	Risperidone (*n* = 190)	Treatment Group Difference	95% CI	Effect size
Primary endpoint: non‐inferiority tests (margin = 7.0)					
PANSS Total Score – mmrm	−31.2 (1.0)	−34.9 (1.0)**	3.7	1.0, 6.3	0.27
PANSS Total Score – ancova	−29.0 (1.2)	−32.0 (1.2)	3	0.0, 5.9	0.18
Secondary endpoints: superiority tests					
PANSS Positive – mmrm	−11.0 (0.32)	−12.2 (0.32)*	1.1	0.3, 2.0	0.27
PANSS Positive – ancova	−10.2 (0.39)	−11.0 (0.40)	0.8	−0.2, 1.8	0.15
PANSS Negative – mmrm	−6.6 (0.33)	−7.5 (0.33)*	0.9	0.0, 1.8	0.20
PANSS Negative – ancova	−6.2 (0.34)	−6.9 (0.34)	0.8	−0.1, 1.6	0.15
CDSS – mmrm	−0.6 (0.08)	−0.5 (0.08)	0	−0.2, 0.2	−0.09
CDSS – ancova	−0.5 (0.11)	−0.4 (0.11)	−0.1	−0.3, 0.2	−0.07
CGI‐S – mmrm	−1.9 (0.07)	−2.1 (0.07)	0.2	−0.0, 0.4	0.21
CGI‐S – ancova	−1.8 (0.08)	−2.0 (0.08)	0.1	−0.1, 0.3	0.18
CGI‐I – mmrm	2.0 (0.06)	1.9 (0.06)	0.1	−0.0, 0.3	0.12
CGI‐I – ancova	2.2 (0.08)	2.1 (0.08)	0.1	−0.1, 0.3	0.09

* *P* < 0.05;

** *P* < 0.01.

Values for CGI‐I are estimated scores at week 6 (LOCF), not change from baseline.

ancova, analysis of covariance; CDSS, Calgary Depression Scale for Schizophrenia; CGI‐I, Clinical Global Impression scale – Improvement; CGI‐S, Clinical Global Impression scale – Severity; MMRM, mixed model repeated measures; PANSS, Positive and Negative Syndrome scale.

The results of the supportive ancova analysis of the PANSS total score were consistent with the mmrm analysis, with a treatment difference of 3.0 points (Table 2). Changes from baseline were statistically significant (*P* < 0.001) in both treatment groups at all post‐baseline assessment time‐points. The majority of patients met responder criteria at endpoint in the lurasidone *versus* risperidone groups (86.1% vs 91.1%), with no significant difference between the groups based on a logistic regression analysis.

Secondary efficacy analyses for superiority yielded results that were largely consistent with the primary (non‐inferiority) analysis of PANSS total score. There were no significant differences between the treatment groups in change from baseline to week 6 for the CGI‐S, CGI‐I, PANSS negative subscale, or CDSS in either the mmrm or ancova analyses (Table 2). As shown in Table 2, between‐group significance was demonstrated at endpoint in favor for risperidone in the mmrm analyses of the PANSS total score and positive and negative subscale scores, however these findings of significance were based on nominal *P*‐values not corrected for multiple comparisons; and, furthermore, were not confirmed based on the supportive ancova analyses of each comparison.

**Figure 2:**
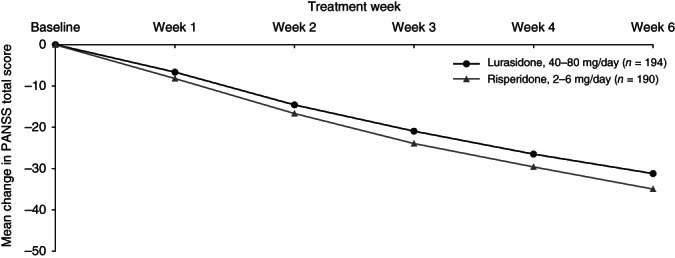
Mean change in PANSS total score.

**Figure 3:**
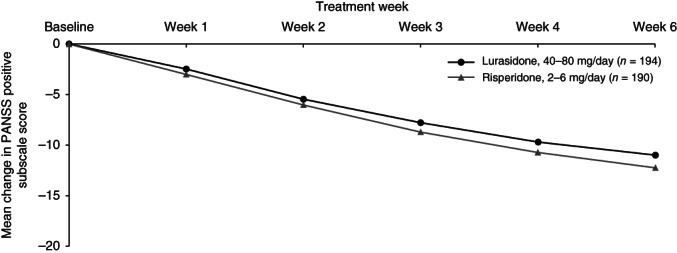
Mean change in PANSS positive subscale score.

**Figure 4:**
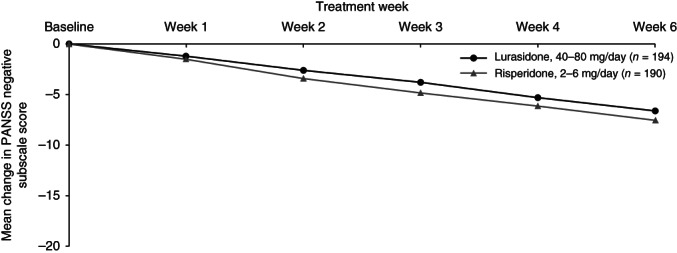
Mean change in PANSS negative subscale score.

**Figure 5:**
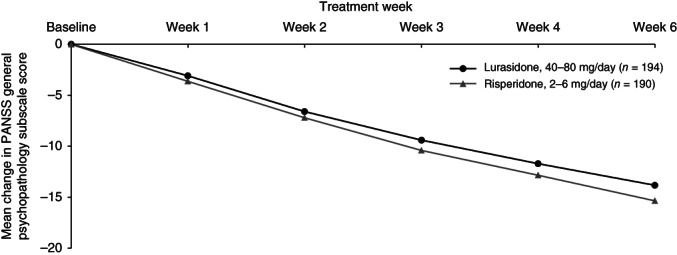
Mean change in PANSS general psychopathology subscale score.

**Figure 6:**
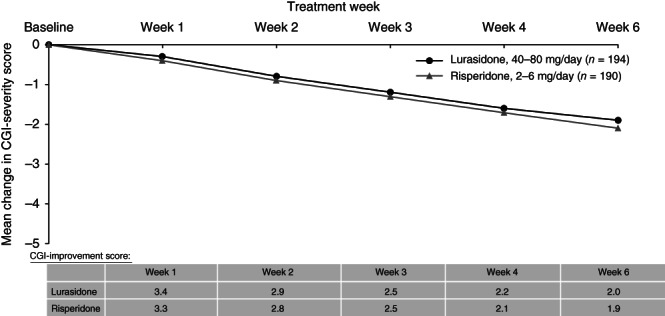
Mean change in CGI‐severity score; Mean CGI‐improvement score.

**Figure 7:**
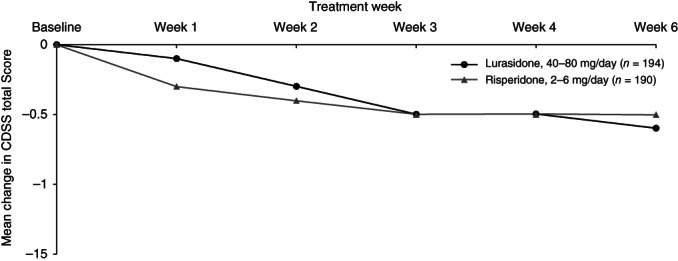
Mean change in Calgary Depression Scale for Schizophrenia (CDSS) score.

Change over time during the 6 weeks of study treatment is displayed in a series of figures for the PANSS total score (Fig. 2), PANSS Positive subscale score (Fig. 3), PANSS Negative subscale score (Fig. 4), PANSS General Psychopathology subscale score (Fig. 5), CGI‐Severity score (Fig. 6; and CGI‐Improvement score), and CDSS total score (Fig. 7).

### Safety

The incidence of treatment‐emergent adverse events was numerically higher in the risperidone group (lurasidone: 69.1%; risperidone: 83.8%; *P* < 0.001; NNH = 7) (Table 3). One serious adverse event (SAE; fracture of the hand) occurred among lurasidone‐treated patients and was judged not related to treatment. No SAEs occurred in the risperidone‐treated patients. No deaths were reported on either drug. Adverse events leading to discontinuation were reported in 2.1% of patients in the lurasidone group and 4.7% of those in the risperidone group. The majority of adverse events were rated as either mild or moderate. Events rated as severe were reported in 2.1% of patients in the lurasidone group and 1.0% of those in the risperidone group.

**Table 3:** Treatment‐emergent adverse events (safety population; frequency ≥ 5%)

Incidence of event, *n* (%)	Lurasidone (*n* = 194)	Risperidone (*n* = 191)	*P*	NNH†
Any adverse event	134 (69.1)	160 (83.8)	<0.001	7
Constipation	25 (12.9)	28 (14.7)	0.614	>50
Nasopharyngitis	19 (9.8)	28 (14.7)	0.145	21
Prolactin increased	6 (3.1)	27 (14.1)	<0.001	10
Insomnia	19 (9.8)	19 (9.9)	0.960	>50
Upper respiratory infection	10 (5.2)	16 (8.4)	0.208	32
Anxiety	12 (6.2)	12 (6.3)	0.969	>50
Weight gain	1 (0.5)	10 (5.2)	0.005	22
EPS‐related events	51 (26.3)	88 (46.1)	<0.001	6
Extrapyramidal disorder	33 (17.0)	73 (38.2)	<0.001	5
Akathisia	14 (7.2)	26 (13.6)	0.040	16

† NNH: number needed to harm; note that treatment with lurasidone was associated with a lower frequency for each adverse event compared to risperidone, therefore lurasidone was used as the reference standard when calculating NNH.

EPS, extrapyramidal symptoms.

EPS‐related adverse events occurred at a lower rate in the lurasidone *versus* risperidone group (26.3% vs 46.1%; *P* < 0.001; NNH = 6; Table 3).

During 6 weeks of study treatment, no clinically meaningful baseline‐to‐endpoint changes, or between‐treatment group differences, were found for vital signs, hematology parameters, HbA_1c_, HDL, LDL, total cholesterol, triglycerides, ECG, or urinalysis parameters. For risperidone *versus* lurasidone, there was significant endpoint increase in glucose (+1.1 mg/dL vs −0.3 mg/dL; *P* < 0.05), serum prolactin (+60.4 ng/mL vs +3.5 ng/mL; *P* < 0.001), and body mass index (+0.45 kg/m^2^ vs +0.20 kg/m^2^; *P* < 0.05). [Correction added on 27 April 2020, after first online publication: Body mass index has been amended.]

Analysis of change from baseline to week 6 (LOCF) on the BARNES, AIMS, and SAS revealed no significant differences between the lurasidone and risperidone groups (+0.2 vs +0.2, *P* = 0.369; +0.0 vs +0.0, *P* = 0.922; +0.5 vs +0.8, *P* = 0.098).

## Discussion

This 6‐week, randomized, double‐blind, non‐inferiority trial evaluated the efficacy and safety of flexibly dosed lurasidone (40 mg/day or 80 mg/day) in comparison to risperidone (2, 4, or 6 mg/day) in patients with schizophrenia in China. On the primary efficacy endpoint, the non‐inferiority of lurasidone relative to risperidone was demonstrated in both the ITT population (primary analysis) and the per‐protocol population. Interpretation of non‐inferiority studies can be difficult because of potential bias in ITT and per‐protocol analyses. However, our confidence in the finding of non‐inferiority is increased due to the consistency of the results for both analysis populations, due to the low overall study discontinuation rate (13.9%; similar for both treatments), and because treatment compliance was similar for the two treatments.

Larger sample sizes are required to perform non‐inferiority testing when compared to sample sizes required in studies designed as superiority trials. Despite the high degree of power associated with large sample sizes used in the current study, superiority testing of secondary efficacy measures revealed no statistically significant between‐treatment group difference, with one exception: there was a small difference favoring risperidone on the PANSS positive subscale. This finding, however, was only evident in the mmrm analysis but not in the ancova analysis. Thus, overall, the results of this study suggest that the efficacy of lurasidone is comparable to risperidone in the treatment of schizophrenia in Chinese patients. Our results are consistent with a recent network meta‐analysis, reporting similar changes in overall symptoms for lurasidone and risperidone in the treatment of schizophrenia.^21^ It should be noted that the meta‐analysis only included studies of patients with acute schizophrenia, while the present study recruited both acute and chronic patients having positive symptoms. Nevertheless, the effect sizes for lurasidone *versus* risperidone on PANSS reported by the two studies were comparable (0.22 vs 0.19).

The safety profile for lurasidone among Chinese patients in the current study was consistent with previous lurasidone studies, with no new safety/tolerability concerns evident. Lurasidone was found to have a more favorable safety profile than risperidone. In particular, there was a lower incidence of EPS and metabolic events, particularly weight increase, and less change in serum prolactin levels, for lurasidone compared to risperidone. These different effects on body weight and prolactin levels replicate those reported in a 12‐month safety trial comparing lurasidone and risperidone,^18^ and are in line with the results of a previous network meta‐analysis.^21^ Our findings are also consistent with the minimal impact on EPS, metabolic parameters, and weight reported for lurasidone in multiple, previous placebo‐controlled trials.^11^, ^12^, ^13^, ^14^, ^15^

The current findings should be understood in light of several limitations of the present study. All sites were in China, and therefore results should not be generalized to patient populations in other countries. Nevertheless, previous studies have yielded similar safety results in other countries.^18^ In addition, study entry criteria excluded patients with concurrent acute medical and psychiatric conditions, which may limit the generalizability of the current results in the subgroup of patients with schizophrenia in the community with medical or psychiatric comorbidity. In addition, both acute and chronic patients having positive symptoms were included, which might influence the results of the efficacy study. Nevertheless, because of the randomized design, this heterogeneity is expected to be balanced between the two groups. Finally, the study duration was too short (6 weeks) to provide an adequate assessment of the effect of study treatment on negative symptoms.

In conclusion, the current study found lurasidone to be non‐inferior to risperidone in terms of antipsychotic efficacy among Chinese patients with schizophrenia. In addition, a more favorable safety profile was evident for lurasidone compared with risperidone, with a lower incidence of EPS, less weight increase, and less change in serum prolactin levels. Thus, lurasidone would appear to offer a useful treatment option for Chinese patients with schizophrenia, with comparable efficacy and improved safety and tolerability consistent with a favorable benefit–risk profile.

## Disclosure statement

The authors declare no conflict of interest.

## Author contributions

All authors were involved in the acquisition and interpretation of the data, and the development and revision of the manuscript; and have approved the final version.

## Supporting information


**Appendix S1.** CONSORT 2010 checklist of information to include when reporting a randomized trial.
